# Treatment of hip dysplasia in patients with mucopolysaccharidosis type I after hematopoietic stem cell transplantation: results of an international consensus procedure

**DOI:** 10.1186/1750-1172-8-155

**Published:** 2013-10-03

**Authors:** Eveline J Langereis, Andrea Borgo, Ellen Crushell, Paul R Harmatz, Peter M van Hasselt, Simon A Jones, Paula M Kelly, Christina Lampe, Johanna H van der Lee, Thierry Odent, Ralph Sakkers, Maurizio Scarpa, Matthias U Schafroth, Peter A Struijs, Vassili Valayannopoulos, Klane K White, Frits A Wijburg

**Affiliations:** 1 Department of Pediatrics and Lysosome Center 'Sphinx’, Academic Medical Center, University of Amsterdam, H7-270, Meibergdreef 9, 1105 AZ Amsterdam, The Netherlands; 2 Department of Orthopaedics and Traumatology, University of Padua, Padua, Italy; 3 National Centre for Inherited Metabolic Disorders, Children’s University Hospital, Dublin, Ireland; 4 Children’s Hospital & Research Center Oakland, Oakland, CA, USA; 5 Department of Pediatrics, Metabolic Diseases, University Medical Center Utrecht, Utrecht, The Netherlands; 6 Genetic Medicine, Manchester Academic Health Science Centre, Central Manchester University Hospitals, NHS Foundation Trust, St Mary’s Hospital, Manchester, UK; 7 Department of Orthopaedic Surgery, Our Lady’s Children’s Hospital, Dublin, Ireland; 8 Department of Pediatric and Adolescent Medicine, Villa Metabolica, University Medical Center of the Johannes Gutenberg University of Mainz, Mainz, Germany; 9 Clinical Research Unit, Woman-Child Center, Academic Medical Center, University of Amsterdam, Amsterdam, The Netherlands; 10 Department of Orthopaedic Surgery, Necker-Enfants/Malades Hospital, Paris Descartes University, Paris, France; 11 Department of Orthopaedic Surgery, University Medical Center Utrecht, Utrecht, The Netherlands; 12 Department of Pediatrics, University of Padua, Padua, Italy; 13 Department of Orthopaedic Surgery, Academic Medical Center, University of Amsterdam, Amsterdam, The Netherlands; 14 IMAGINE Institute and Paris Descartes University, Reference Centre for Inherited Metabolic Diseases, Necker-Enfants Malades Hospital, Paris, France; 15 Department of Orthopedics and Sports Medicine, Seattle Children’s Hospital, University of Washington, Seattle, WA, USA

**Keywords:** Mucopolysaccharidosis type I, Hurler syndrome, Hematopoietic stem cell transplantation, Dysostosis multiplex, Hip dysplasia, Surgical treatment, Consensus

## Abstract

**Background:**

Mucopolysaccharidosis type I (MPS-I) is a lysosomal storage disorder characterized by progressive multi-organ disease. The standard of care for patients with the severe phenotype (Hurler syndrome, MPS I-H) is early hematopoietic stem cell transplantation (HSCT). However, skeletal disease, including hip dysplasia, is almost invariably present in MPS I-H, and appears to be particularly unresponsive to HSCT. Hip dysplasia may lead to pain and loss of ambulation, at least in a subset of patients, if left untreated. However, there is a lack of evidence to guide the development of clinical guidelines for the follow-up and treatment of hip dysplasia in patients with MPS I-H. Therefore, an international Delphi consensus procedure was initiated to construct consensus-based clinical practice guidelines in the absence of available evidence.

**Methods:**

A literature review was conducted, and publications were graded according to their level of evidence. For the development of consensus guidelines, eight metabolic pediatricians and nine orthopedic surgeons with experience in the care of MPS I patients were invited to participate. Eleven case histories were assessed in two written rounds. For each case, the experts were asked if they would perform surgery, and they were asked to provide information on the aspects deemed essential or complicating in the decision-making process. In a subsequent face-to-face meeting, the results were presented and discussed. Draft consensus statements were discussed and adjusted until consensus was reached.

**Results:**

Consensus was reached on seven statements. The panel concluded that early corrective surgery for MPS I-H patients with hip dysplasia should be considered. However, there was no full consensus as to whether such a procedure should be offered to all patients with hip dysplasia to prevent complications or whether a more conservative approach with surgical intervention only in those patients who develop clinically relevant symptoms due to the hip dysplasia is warranted.

**Conclusions:**

This international consensus procedure led to the construction of clinical practice guidelines for hip dysplasia in transplanted MPS I-H patients. Early corrective surgery should be considered, but further research is needed to establish its efficacy and role in the treatment of hip dysplasia as seen in MPS I.

## Background

Mucopolysaccharidosis type I (MPS I) is an autosomal recessive lysosomal storage disorder caused by a deficiency of the lysosomal hydrolase α-L-iduronidase (IDUA) [1]. The progressive accumulation of the glycosaminoglycans (GAGs) heparan sulfate and dermatan sulfate in virtually all body tissues leads to progressive multisystem disease. MPS I encompasses a wide phenotypic spectrum, with the attenuated end of this spectrum (Scheie syndrome; MPS I-S) characterized by progressive musculoskeletal, pulmonary and cardiac disease and a relatively normal life expectancy. On the other end of the spectrum is severe Hurler syndrome (MPS I-H), which is the most prevalent phenotype, with progressive central nervous system (CNS) disease in addition to generally more severe somatic manifestations, resulting in a significantly reduced life expectancy if left untreated [2].

**Figure 1:**
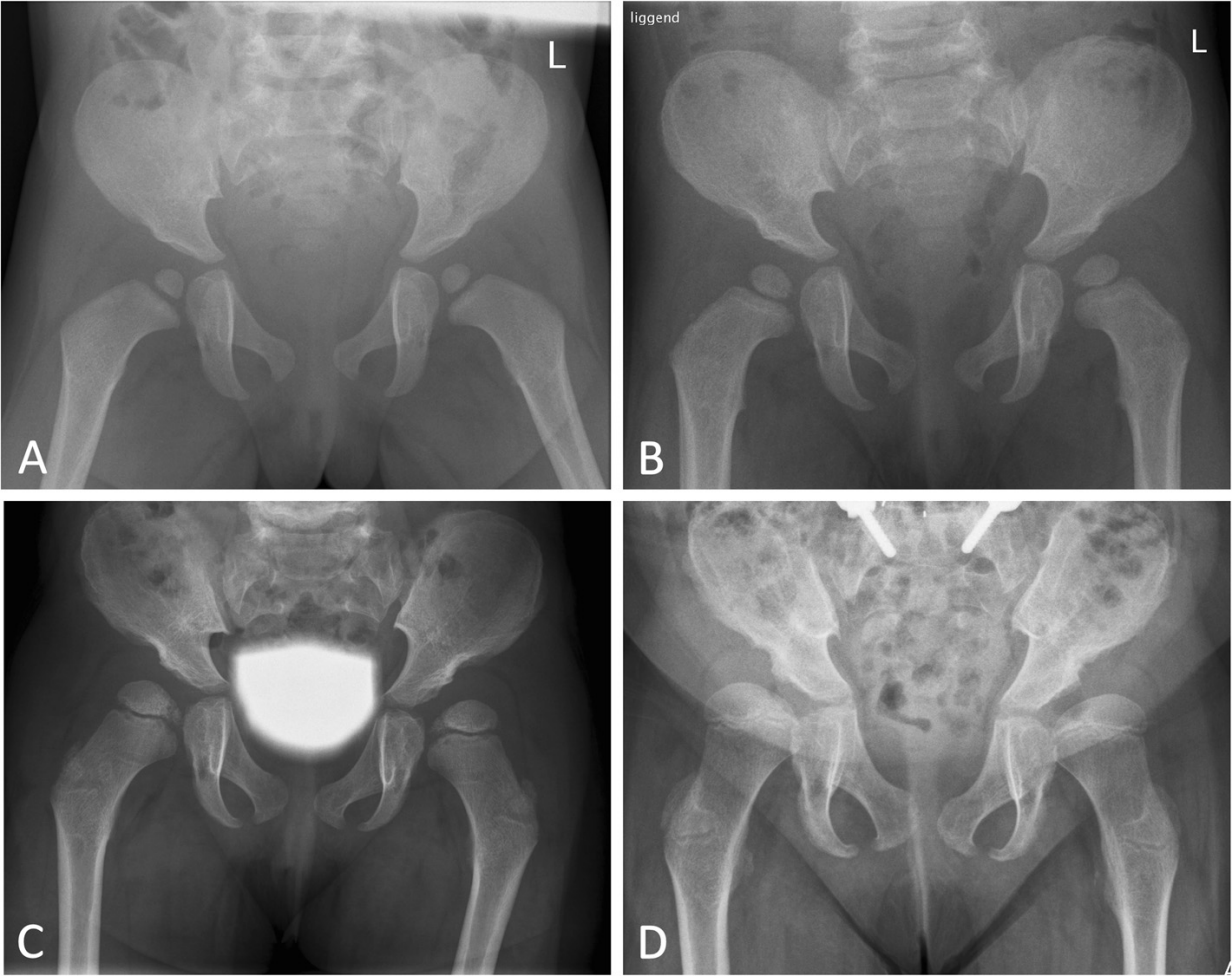
**Sequential X-ray studies of a MPS I-H patient who underwent successful HSCT at the age of 2 yrs and 6 m as well as spinal fusion at the age of 7.** Characteristic signs included acetabular dysplasia with a steep acetabular angle, interruption of Shenton’s line, characteristic medial thinning of the femoral head and coxa valga. **A**: at diagnosis, age 1 yrs 3 m **B**: at 2 yrs 4 m **C**: at 4 yrs 8 m **D**: at 8 yrs 5 m.

The skeletal disease associated with MPS I is generally referred to as 'dysostosis multiplex’ , a collection of radiographic abnormalities resulting from defective endochondral and membranous growth throughout the body [3-5]. Typically, the growth of the long bones is stunted, vertebral bodies are hypoplastic, which may result in kyphosis with or without scoliosis, and the knees are in the valgus position. Hip abnormalities, due to failure of ossification of the lateral acetabular roof, medial proximal epiphyseal growth failure of the femur and coxa valga, lead to a complex form of hip dysplasia that is often accompanied by deformation, subluxation or dislocation of the femoral head (Figure 1). Other findings include bullet-shaped metacarpals and phalanges, an enlarged and thickened skull, broad clavicles and broad oar-shaped ribs [6,7]. The pathophysiology of the skeletal disease in MPS I, as in the other mucopolysaccharidoses, is complex and not fully understood. Intra- and extracellular deposition of GAGs leads to impaired cell-to-cell signaling, altered mechanical properties and upregulated inflammatory pathways, which are all believed to affect the growth plate, osteoclasts and osteoblasts while contributing to the typical bone pathology [8,9]. Furthermore, accumulation of GAGs in the soft tissues and the consequent pathological cascade may contribute to joint stiffness and limited mobility.

While intravenous enzyme replacement therapy (ERT; recombinant IDUA, laronidase) is indicated for the treatment of the non-neurological manifestations of MPS I [10], hematopoietic stem cell transplantation (HSCT) is the treatment of choice for patients with the MPS I-H phenotype. In contrast to ERT, HSCT can preserve cognitive function in addition to ameliorating many of the somatic symptoms of MPS I [11,12]. Due to the progressive nature of the CNS disease, HSCT should be performed at an early stage of the disease, preferably before the age of 2.5 years [10].

Although HSCT has shown favorable effects related to several important clinical outcome parameters, the skeletal disease is particularly unresponsive to this treatment, with a variable progression of genu valgum, thoracolumbar kyphosis and hip dysplasia [3,13-19].

Several studies have shown that hip dysplasia is very common in patients with MPS I-H, even following successful HSCT, and that it generally has a progressive course, as observed on sequential radiographic studies [3,13-19]. Advanced hip disease in successfully transplanted MPS I-H patients may lead to pain and functional complaints in adolescence or early adulthood, at least in a subset of patients, as reported in a number of case series [3,14].

There are two approaches to treat hip dysplasia associated with MPS I: 1) early corrective surgery and 2) surgery to treat clinical symptoms such as pain and functional disability. First, early corrective osteotomies (e.g., Salter, Dega or Pemberton innominate osteotomies, with or without femoral varus osteotomies [20-22]), which aim to correct the anatomical abnormalities seen in these patients, can be performed when the femoral head and acetabulum are still sufficiently congruent. Because of the lack of sufficient remodeling potential of the femur and acetabulum, these procedures are best performed before the age of 6–7 years or earlier to attempt to prevent progressive deformity. Second, salvage procedures (e.g., a shelf augmentation or Chiari osteotomy [23]), can be performed at a later age when corrective osteotomies are not feasible, as these procedures do not require congruent hips. The main indication for these salvage procedures is to reduce pain from subluxation, and to increase bone stock for future hip replacement.

As HSCT is increasingly successful due to improved conditioning regimens and different stem cell sources, over 80% of patients now remain alive and engrafted with a significantly improved life expectancy [24,25]. However, there is a paucity of data regarding which successfully transplanted patients will develop hip dysplasia, which patients will develop symptoms of hip dysplasia, such as pain and impaired locomotion, and when surgical intervention is needed in patients with hip dysplasia. There is thus a need for consensus-based guidelines. To develop such guidelines in the absence of evidence, we initiated an international expert consensus procedure and used a modified Delphi technique, with the aim to provide consensus-based treatment recommendations on hip dysplasia in MPS I patients.

## Methods

A modified Delphi technique was used to explore expert opinions and obtain a consensus where possible, as this method recognizes the value of experts’ opinions, experience and intuition when full scientific knowledge is lacking [26].

As a first step in the procedure, a literature review was performed by one of the researchers (EJL) to identify all published material on the treatment strategies used in MPS I patients with hip disease. A search was conducted in the electronic databases of PubMed (http://www.pubmed.gov) and EMBASE (http://www.embase.com) using the key words 'mucopolysaccharidosis type I; MPS I; hip; dysplasia; treatment’. Papers were included when they described MPS I patients and contained descriptions of treatment (ERT or HSCT), prevalence of hip dysplasia in the reported cohort, treatment strategies for the hip dysplasia and outcomes of the intervention. Papers were excluded if only the prevalence of hip dysplasia was reported without data on the clinical course, intervention or outcome. Only original papers reporting on patients cohorts were included. The selected papers were summarized with a focus on the outcomes of surgery; this overview was presented and discussed during the face-to-face meeting. Eight metabolic pediatricians (MB, EC, PRH, PMvH, SAJ, MS, VV, FAW) and nine orthopedic surgeons (AB, AF, PMK, CL, TO, RJS, MUS, PAS, KKW) were invited to participate in the Delphi panel. All invitees had significant experience in the treatment of patients with MPS I. When possible, an orthopedic surgeon and a pediatrician were invited from the same center. In addition, one orthopedic surgeon specializing in the treatment of adult patients with lysosomal storage disorders and hip replacement surgery was invited (MUS).

The procedure consisted of two written rounds and a face-to-face meeting. For the written rounds, 11 case histories were gathered from two participating centers (Manchester, UK; Amsterdam, the Netherlands), covering both the severe and milder forms of hip abnormalities as well as all MPS I phenotypes. For each patient, clinical information on diagnosis (age, clinical symptoms) and treatment (ERT or HSCT, time of initiation) was provided. For each case, the clinical course was reported, with a focus on psychomotor development, mobility and pain. Sequential radiographic images (X-ray, CT or MRI) were provided. If surgery had been performed, only the clinical course and radiographic images prior to the surgical intervention were reported, and the decision whether to operate was not reported. The case series included nine MPS I-H patients, one MPS I-S patient and one patient with the intermediate Hurler-Scheie phenotype.

In all written rounds, the panel members received the cases in random order together with a survey asking for each case whether they would choose to perform surgery (yes / no / might consider but need more information). In addition, in the first written round, the experts were asked for each case description to state essential aspects leading to the decision and issues complicating the decision making. These open-ended responses were subsequently categorized by two independent investigators (JvL, EL).

Draft statements on the optimal approach to hip dysplasia in MPS I patients, based on the literature review and clinical experience, were composed by three of the authors (FAW, EJL, SAJ).

Approximately one month after the first written round, a face-to-face meeting was held in Amsterdam, the Netherlands. This meeting was chaired by an independent moderator (JvL) not involved in the treatment of patients with MPS I. Before beginning the actual meeting, the second written round was performed. All 11 cases were again presented to the participants (in a randomly different order), and they were asked whether they would choose to perform surgery (yes / no / might consider but need more information). Statistical analyses for intra-observer variation and reliability of agreement were performed using SPSS 19.0. Intra-observer reliability was quantified for every specialist using Cohen’s kappa [27], whereas the inter-rater reliability was assessed using Fleiss’ kappa; both of these are measures of agreement that correct for agreement by chance. Kappa values may vary from ≤ 0 (complete disagreement, besides expected agreement based on probability) to 1 (complete agreement). Generally, a kappa value of 0.61-0.80 is considered to indicate “good agreement” [28]. This second written round was followed by detailed discussions concerning the most appropriate treatment approach for each patient, with a particular focus on areas of controversy. These discussions were fuelled by the information on issues complicating decision making, as given during the first written round. The aim of this discussion was to gather all relevant issues related to the decision-making processes concerning treatment decisions.

In the second phase of the face-to-face meeting, the draft consensus statements were discussed and revised until a full consensus was reached on each of the statements. This resulted in the proposed recommendations for follow-up and treatment of patients with hip pathology due to MPS I-H.

## Results

The literature search resulted in 14 papers related to hip dysplasia in MPS I. Six of these papers evaluated the management of hip dysplasia in MPS I and were further analyzed. The maximum recorded follow-up duration was 19 years post-HSCT. In two manuscripts, there was overlap in the reported patients, and one paper did not indicate the number of surgeries performed. The remaining four papers reported on 56 patients; the outcomes of surgery for hip dysplasia in 28 patients were reported (Table 1).

**Table 1:** Summary of literature on the treatment of hip dysplasia in transplanted MPS I-H patients

**First author (year of publication)**	**Patients with hip dysplasia (total patients reported)**	**Surgical interventions (n/o patients)**	**Mean age at surgery (years)**	**Mean follow-up after surgery (years)**	**Radiological outcome**	**Functional outcome**
Field (1994)	11 (11)	Femoral osteotomy (?)	-	-	No acetabular remodeling	-
Souillet (2003)	11 (15)	Acetabular and femoral osteotomy (1)	7.4	1.4	No dislocation	-
		Femoral osteotomy (7)				
		Total hip replacement (1)				
Weisstein (2004)	6 (7)	Acetabular and femoral osteotomy (4)	4.8	-	Mean acetabular angle from 38° to 20°	Normal range of motion in 4/6
		Pelvic osteotomy (2)			Appropriate femoral head coverage	
Taylor (2008)	23 (23)	Acetabular and femoral osteotomy (8)	4.4	8.0	Mean acetabular angle from 34° to 22°	Good range of motion
		Acetabular osteotomy (4)			Mean center edge-angle 40° (long term)	Number of older patients complaining of hip discomfort
		Femoral osteotomy (1)				

All 17 invited specialists accepted the invitation and took part in the first written round. One pediatrician and one orthopedic surgeon from two different centers were unable to attend the face-to-face meeting due to severe weather conditions and disruption of air travel, so 15 specialists participated in the face-to-face meeting and the second written round. Tables 2 and 3 show the four most commonly reported items considered to be important for, or complicating, the decision-making process.

**Table 2:** The four most frequently reported aspects that were considered essential for the surgical decision-making process

	**Decision to perform surgery**		
	**Yes**	**No**	**Might consider**
**Essential aspects**	Radiological features (progression)	Absence of pain	Radiological features
	Presence of pain	Absence of disability	Absence/presence of pain
	Presence of disability	Radiological features (stable course)	Absence/presence of disability
	Expected progression of symptoms	Stable clinical course so far	Expected progression of symptoms

**Table 3:** The four most frequently reported aspects complicating surgical decision making

	**Decision to perform surgery**		
	**Yes**	**No**	**Might consider**
**Complicating aspects**	Radiological features (complicating surgery)	Radiological features without pain	Radiological features
	Absence of pain	Lack of evidence	Absence/presence of pain
	Progression of symptoms so far	Absence of disability	Unclear prognosis with/without surgery
	Expected progression of symptoms	Stable clinical course so far	Absence/presence of disability

Fleiss’ kappa as a measure for inter-rater reliability was 0.19 and 0.13 in the first and second rounds respectively, indicating that consensus amongst the specialists had not changed before the face-to-face meeting The median [range] Cohen’s kappa, indicating intra-rater reliability between the first and second round, was 0.43 [-0.16 to 0.72].

**Figure 2:**
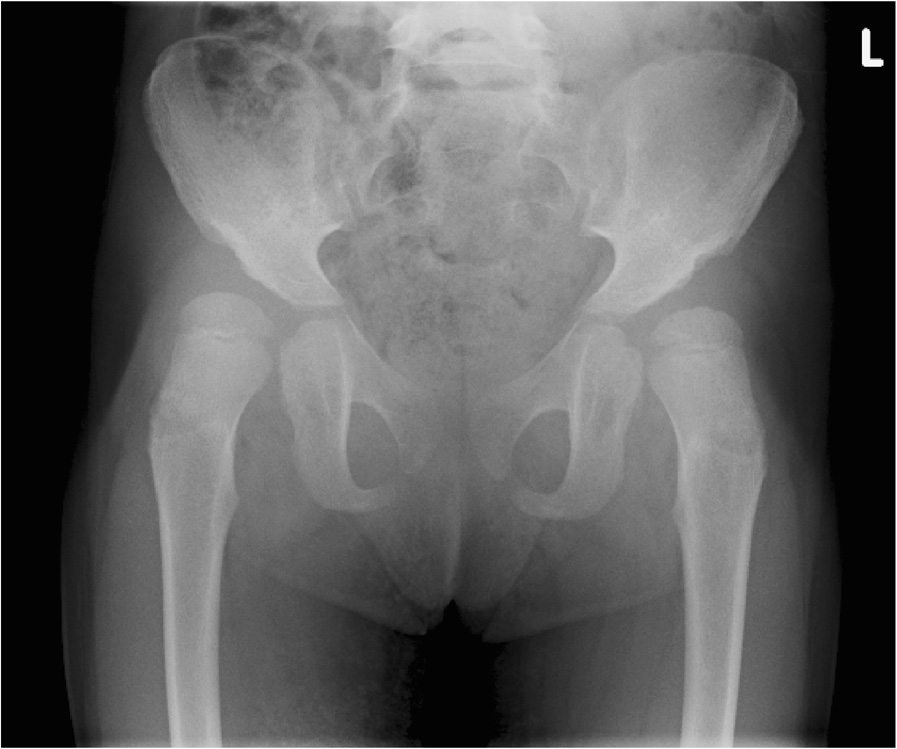
Pelvic X-ray at age 4 years.

The following case illustrates the consensus procedure. Case: Female MPS I patient, diagnosed at the age of 8 months. Presenting symptoms included respiratory tract infections and course facial features. She was treated with ERT (Aldurazyme®) for two months prior to successful haematopoietic stem cell transplantation at age 13 months. Currently, at the age of 4 years, she shows a delay in motor and cognitive development. She has a normal range of motion, reasonable locomotion with a wide gait. She is able to run and climb, with some mild complaints of tiredness in both legs (Figure 2).

Participants responded to the question “Considering the provided clinical and radiological data, would you choose to perform surgery in this patient?” as follows: No 47%, Yes 29%, Maybe 24%.

Highlights from the discussion:

• For 80% of specialists who favoured surgical intervention, the appearance and progression of radiological features was an argument for their decision.

• For 62% of the specialists who were against surgical intervention, the absence of complaints was an argument for their decision.

• In the face-to-face meeting the focus of the discussion was whether future complaints were expected or not. Specialists in favour of surgical intervention all expected complaints, specialists who favoured a conservative approach argued that severity of radiological features did not match (future) clinical complaints.

• Early intervention was thought to improve changes of orthopaedic success and to improve anatomical outcomes for later interventions

• No consensus was reached on this case

During discussion of the case histories and the draft statements, it became clear to all participants that obtaining a consensus and formulating clinical guidelines for the treatment of hip dysplasia for all MPS I phenotypes was not feasible due to the broad phenotypic spectrum of MPS I, the rarity of patients with more attenuated phenotypes and the complete lack of published case reports on more attenuated patients. As the severe MPS I-H phenotype is the most prevalent of the MPS I phenotypes and hip dysplasia appears to be uniformly present in MPS I-H, it was decided to limit the recommendations to transplanted MPS I-H patients; from then on, only case histories of MPS I-H patients (nine of the 11 case histories) were further discussed.

Full consensus was reached on the following seven statements.

1. Hip dysplasia is a very common symptom in MPS I-H patients. The anatomical abnormalities are generally progressive, even after successful HSCT, and may result in significant morbidity and functional impairment.

HSCT cannot prevent or stabilize progression of the hip abnormalities in most patients [3,17,18,29]. Although data on the long-term follow-up of transplanted MPS I-H patients are very limited, the radiological characteristics, such as (sub)luxation of the hips and decreased articular cartilage, suggest that hip dysplasia may ultimately result, at least in a subgroup of patients, in pain and loss of function.

2. The rate of progression of hip dysplasia in successfully transplanted MPS I-H patients varies, and there are no valid methods to predict its progression or the risk for development of symptoms attributable to MPS I-H-related hip dysplasia.

Because the pathophysiology of hip dysplasia in MPS I-H patients differs from the relatively common developmental dysplasia of the hip (DDH), data on the course of the disease in DDH cannot be used to predict the course of the hip dysplasia in transplanted MPS I-H patients. In addition, the severity of hip abnormalities as assessed by radiographic studies varies significantly within the transplanted Hurler population [3,13-19].The risk for pain and functional disability is likely to be correlated with the severity of abnormalities (i.e., (sub)luxation of the femoral head, steep acetabular angle) at an early age. However, other factors such as age at transplantation, donor chimerism, extent of somatic and neurological residual disease and overall mobility may also influence the clinical course and therefore the risk for the development of pain.

3. The presence and severity of hip abnormalities should be assessed soon after the diagnosis of MPS I in all patients. This should be done according to radiographic studies and include at least an AP pelvic X-ray study.

Although predicting the clinical course of hip abnormalities is currently very difficult, or even impossible, in individual patients with MPS I-H, early assessment of hip abnormalities is important for the early identification of patients with hip dysplasia. Future treatment decisions can then be based upon serial radiographic studies, allowing assessment of the rate of progression.

4. Patients with MPS I-H should be followed up regularly from the time of diagnosis by a multi-disciplinary team. This team should include a pediatric orthopedic surgeon.

A multi-disciplinary team composed of specialists from various medical disciplines and including other healthcare professionals such as physiotherapists is needed for optimal care during the follow-up of MPS I-H patients [30]. To allow for early discussions on the optimal treatment strategy in individual patients and timely surgery, a pediatric orthopedic surgeon should be a member of the multidisciplinary MPS I team rather than only available upon consultation.

5. Hip surgery should be considered as a treatment option in all transplanted MPS I-H patients with hip dysplasia.

Early reconstructive surgery is primarily aimed at the prevention of complications (pain, dislocation) later in life and needs to be performed at a relatively early age, before deformation of the femoral head. This approach was considered optimal by a majority, but not all of the panelists. As not all patients will develop pain and/or hip dislocation, specialists may also choose to monitor the hip dysplasia and only consider surgery when symptoms develop that interfere with the patient’s quality of life. As this generally occurs later in life, reconstructive surgery will often not be feasible, and a salvage procedure, aimed at stabilizing the femoral head to prevent full dislocation, will then be the treatment of choice. This latter approach was considered optimal by a minority of the panelists.

As life expectancy of transplanted MPS I patients has increased considerably and total hip replacement is probably an important future treatment option for osteoarthritis of the hips in adult patients both early corrective surgery (e.g., by a Salter, Dega or Pemberton procedure) and late salvage surgery should aim for creating sufficient bone stock for later hip replacement surgery.

Patients with chronic pain due to advanced hip disease and for whom a salvage procedure or a total hip replacement is not feasible due to anatomical deformation or other complicating factors should be treated with chronic pain medication or by a resection arthroplasty of the hips (Girdlestone procedure).

6. If a patient expresses pain that is likely located in the hips, it is important to try to establish whether the pain is caused by arthropathy.

Chronic hip pain due to arthropathy that impacts the patient’s quality of life or necessitates chronic pain medication can be an indication for hip surgery. However, it can be difficult to assess whether the pain is indeed caused by arthropathy, especially in children. Even after successful transplantation, MPS I-H patients may have significant residual disease, such as lumbar kyphosis, myelopathy, joint stiffness and genu valgum, which may impair locomotion and result in hip pain. An intra-articular injection of local anesthetics may be considered as a tool to establish whether the pain is caused by intra-articular pathology.

7. For all patients for whom surgical intervention is considered, a number of factors need to be taken into account. These include life expectancy, neurological status, musculoskeletal symptoms, expected rehabilitation course, general condition, anesthetic risks, expected mobility with or without surgery and quality of life.

Decisions regarding hip surgery in transplanted MPS I-H patients need to be carefully balanced, and a number of factors need to be taken into account. Despite the considerably improved outcome of HSCT, life expectancy in successfully transplanted MPS I-H patients may still be reduced due to residual MPS I-H-related disease. This should be taken into account when surgery aimed at the prevention of long-term complications like arthritis is considered. A more conservative approach, e.g., long-term use of analgesics, may also be considered. In addition, once full dislocation has occurred, this may reduce or even fully abolish the pain. Furthermore, neurological and musculoskeletal disease may significantly complicate the final functional outcome of hip surgery. Some patients may lose their ability for independent locomotion and may become fully wheelchair-bound before adulthood due to causes other than hip disease.

Other MPS I-H-related symptoms, such as cognitive impairment, may also impact the potential for rehabilitation after surgery. Finally, a number of other MPS I-H-related issues, such as spinal deformity, upper airway disease, cardiomyopathy or restrictive lung disease [31], may result in increased anesthetic and perioperative risks.

## Discussion

This international consensus procedure was initiated to develop clinical practice guidelines for the management of hip dysplasia in patients with MPS I. Because of the broad phenotypic spectrum of MPS I, the high prevalence of hip dysplasia in MPS I-H patients and the lack of published cases or case series on patients with a more attenuated phenotype, it was decided to focus on MPS I-H patients during the consensus meeting. The considerable increase in life expectancy of MPS I-H patients due to the improved outcome of HSCT has revealed that many patients suffer from progressive, residual orthopedic disease [3,14-19,32], which underpins the need for the development of practice guidelines. Ideally, best practice guidelines are based upon systematic reviews (level 1 evidence; Oxford Centre for Evidence Based Medicine; http://www.cebm.net) or prospective, randomized controlled trials (level 2 evidence). Randomized controlled trials are, however, extremely difficult to perform in a very rare and heterogeneous disease such as MPS I-H. Additionally, such studies are further complicated by the very long follow-up period of at least 20 years needed to assess the effects of any intervention, compared to a more conservative wait-and-see-approach for hip dysplasia. At present, there are only case series on the surgical management of hip dysplasia reported in the literature (level 4 evidence, Table 1), and these studies do not report on the effects of surgery on long-term complications such as osteoarthritis [3,15-18].

In the absence of sufficient data to make an evidence-based clinical practice protocol, we decided to use the Delphi technique, as this allows the combination of available evidence with expert opinion to develop guidelines. Structured feedback on the responses gathered then allows for a focused discussion and leaves room for a variety of opinions.

Full consensus was reached on a number of important issues. However, there was no full consensus as to the essential question of whether early corrective hip surgery is the optimal treatment for all transplanted MPS I-H patients with hip dysplasia on radiographic studies. Most experts preferred early corrective surgery to prevent progressive hip subluxation and subsequent arthrosis. Since not all patients develop hip pain or functional disabilities as a consequence of hip dysplasia, at least not before early adolescence, a minority of panel members opted for a more conservative approach. They elected surgical intervention only if chronic hip pain, due to the misalignment of the femoral head and acetabulum, develops. In the absence of data on both the natural course of hip dysplasia in MPS I-H without surgical intervention and on the long-term outcome of early corrective surgery in transplanted MPS I-H patients, it is recommended that decisions on treatment should be based on evaluation by a multi-disciplinary team, weighing the pros and cons of early corrective surgery.

The current study had several limitations. First, the Delphi technique was developed to explore expert opinions and converge these towards a “mean”, which might suggest a scientific truth, which is not evidence based. Additionally, despite convergence, the influence of “strong personalities” cannot be fully overcome. Potential “molding of opinions” by the investigators was addressed by the choice of an independent moderator [33].

Second, there were two deviations from the initial study design. The inability of two participants to attend the face-to-face meeting led to a lower number of responders. Additionally, the decision to focus on MPS I-H patients rather than on discussing treatment options for the full phenotypic spectrum left some patient cases redundant.

This consensus procedure resulted in a framework of clinical practice guidelines that can be used in the follow-up and management of hip dysplasia in patients with MPS I-H. Despite this narrowed focus, we feel that many of the statements might also be applicable for Hurler/Scheie or Scheie patients with severe hip dysplasia. Furthermore, it must be stressed that clinical practice guidelines can never replace the physician’s clinical judgment. Future research on the natural history of hip dysplasia in all MPS I phenotypes and on the long-term outcomes of early surgical intervention is much needed but difficult to perform. Focusing on patient quality of life and functional outcomes is essential, considering the variability of residual disease seen in transplanted MPS I-H patients, and the general lack of correlation between radiology findings and patient reported outcomes.

## Conclusions

This international consensus procedure led to the construction of clinical practice guidelines for hip dysplasia in transplanted MPS I-H patients. Early corrective surgery should be considered, but further research is needed to establish its efficacy and role in the treatment of hip dysplasia as seen in MPS I.

## Abbreviations

AP X-ray: Anteroposterior X-ray; CNS: Central nervous system; CT: Computed tomography scan; DDH: Developmental dysplasia of the hip; ERT: Enzyme replacement therapy; GAGs: Glycosaminoglycans; HSCT: Hematopoietic stem cell transplantation; IDUA: α-L-iduronidase; MPS I: Mucopolysaccharidosis type I; MPS I-H: MPS I-Hurler; MPS I-S: MPS I-Scheie; MRI: Magnetic resonance imaging.

## Competing interests

The authors declare that they have no competing interests.

## Authors’ contributions

EJL, FAW, JvL: Conception and design, data acquisition, analysis and interpretation, manuscript draft and revision. AB, EC, PRH, PMvH, SAJ, PMK, CL, TO, RJS, MS, MUS, PAS, VV, KKW, FAW: Full participation in the written rounds and the face-to-face meeting of the consensus procedure, revision of the manuscript. All authors read and approved the final manuscript.
